# Indications of Clinical Application of L5 Laminar Hook for the Surgical Correction of Degenerative Sagittal Imbalance

**DOI:** 10.3390/medicina61111963

**Published:** 2025-10-31

**Authors:** Xiongjie Li, Yong-Chan Kim, In-Seok Son, Sung-Min Kim

**Affiliations:** Department of Orthopaedic Surgery, Kyung Hee University Hospital at Gangdong, College of Medicine, Kyung Hee University, Seoul 05278, Republic of Korea; lixiongjie223@gmail.com (X.L.); yckimspine@gmail.com (Y.-C.K.); wiselove@naver.com (I.-S.S.)

**Keywords:** L5 laminar hook, adjacent segmental degeneration, sagittal alignment, surgical outcome, adult spinal deformity

## Abstract

*Background and Objectives*: The selection of the optimal distal level of fusion in long-level fusion surgery remains controversial. Fusion ending at L5 preserves motion but increases the risk of L5–S1 disc degeneration. The L5 laminar hook has been introduced to enhance distal fixation, but its indications and clinical effects remain unclear. This study aimed to investigate the indication of the L5 laminar hook and its effect on adjacent segment degeneration when performing long-level fusion terminating at L5 in patients with degenerative sagittal imbalance (DSI). *Materials and Methods*: 112 patients with DSI who had undergone corrective surgery and ended at L5 were analyzed retrospectively. Finally, 64 were treated with an L5 laminar hook (Group I), while 48 were not (Group II). We compared radiographic spinopelvic parameters between the two groups and followed up disc degeneration at the L5–S1 level. Additional analysis was conducted within Group I to determine the indications for L5 laminar hook. *Results*: Preoperative C7 sagittal vertical axis (C7SVA), lumbar lordosis (LL), and pelvic incidence (PI)-LL mismatch were significantly greater in Group I than in Group II (*p* < 0.05). At 2 years of follow-up, advanced L5–S1 disc degeneration had developed in 14 patients (21.9%) in Group I and 36 patients (75%) in Group II. Patients in Group B (exacerbated L5–S1) had a significantly higher body mass index (BMI), larger preoperative C7SVA, and PI-LL mismatch than patients in Group A (preserved L5–S1). The C7SVA and PI-LL mismatch cut-off values for Group A were 15.8 cm and 40.8°, respectively. *Conclusions*: L5 laminar hook helps to reduce disc degeneration at the L5–S1 level and can be used to maintain the deformity correction ending at L5 in patients with DSI. We propose a series of indicators for the use of L5 laminar hooks in patients with DSI: a low BMI, a PI-LL mismatch < 40.8°, and a C7SVA < 15.8 cm.

## 1. Introduction

There is currently no consensus of opinion relating to whether instrumentation should be stopped at L5 or S1 when performing long-level fusion in patients with adult spinal deformity [1]. When fusion is stopped at L5, postoperative complications may include degeneration of adjacent segments, especially at L5–S1 disc level, which may lead to radiculopathy or distal junctional failure [2]. Finally, patients with complications will require additional L5–S1 spinal fusion with pelvic fixation [1,2].

Laminar hooks have been used to facilitate the distal junction of long-level instrumentation since the 1990s [3]. Existing publications report that laminar hook can be applied without fluoroscopic control, are associated with less blood loss than other techniques, and can maintain a stability similar to that of a pedicle screw system [4]. In addition, experimental research by Hasegawa et al. demonstrated that the combination of a pedicle screw system and laminar hook can achieve firm fixation when used to treat an osteoporotic spine [5]. However, do date, there has been no study on how laminar hook affects the adjacent segment degeneration.

In this study, we aimed to investigate the clinical indication of the L5 laminar hook and its effect on adjacent segment degeneration when performing long-level fusion terminating at L5 in patients with degenerative sagittal imbalance (DSI).

## 2. Materials and Methods

### 2.1. Study Design and Participants

This was a retrospective analysis of patients with DSI who underwent surgery to correct spinal deformities at our institution. Institutional Review Board approval was granted for this study to proceed (KHNMC 2024-04-007). We reviewed 265 patients with DSI who underwent deformity corrective surgery performed by a single surgeon between March 2017 and June 2019. The inclusion criteria were as follows: (1) age ≥ 60 years; (2) preoperative Pfirrmann grade [6] ≤ 3 at L5–S1 segment; (3) upper instrumented vertebra (UIV) at ≥T12 and lower instrumented vertebra (LIV) at L5; and (4) a minimum follow-up period of ≥2 years. The exclusion criteria were as follows: (1) sacralization of L5, (2) a previous history of L5–S1 surgery; (3) diagnosed with degenerative lumbar scoliosis; and (3) gait impairment due to a musculoskeletal disease or congenital anomalies. Finally, only 112 patients were included in this study. The patients were divided into two groups: Group I (applied with laminar hook) and Group II (not applied with laminar hook). For further analysis to determine the indication of L5 lamina hook, Group I patients were divided into the following two groups: Group A (preserved L5–S1 disc) and Group B (exacerbated L5–S1 disc).

### 2.2. Radiographic Analysis

Preoperative and postoperative (immediate and 2 year postoperative) standing lateral radiographs of the whole spine were retrieved and viewed using the ZeTTA PACS Viewer version 2.0.2.6 (TaeYoung Soft Co., Ltd., Gwacheon-si, Republic of Korea). We then measured a range of radiographic sagittal spinopelvic parameters: C7 sagittal vertical axis (C7SVA), thoracic kyphosis (TK), thoracolumbar kyphosis (TLK), lumbar lordosis (LL), pelvic tilt (PT), sacral slope (SS), pelvic incidence (PI), and PI-LL mismatch.

### 2.3. Disc Degeneration Assessment

The status of the lumbosacral discs (L5–S1) on magnetic resonance imaging (MRI) was evaluated using the Pfirrmann. Based on a previous study by Cannizzaro et al. [7], we classified Pfirrmann grade 1, 2, and 3 as ‘healthy disc’ and grade 4 and 5 as ‘degenerated disc’. Disc status was re-evaluated 2 years postoperatively.

### 2.4. Radiographic Parameters and Disc Status Measurement

All radiographic parameters and disc status were independently collected by two spine surgeons who were not involved in related operations. The surgeons measured all data for a second time, with an interval of one week. The inter-rater and intra-rater reliabilities were calculated using kappa statistics. The intraclass correlation coefficient (ICC) was measured to assess agreement between observers [8,9].

### 2.5. Statistical Analysis

All statistical analyses were performed using SPSS version 21.0 (IBM Corp., Armonk, NY, USA). The Shapiro–Wilk test was used to assess data normality. Continuous variables were expressed as mean ± standard deviation, and categorical variables as number and percentage. Between-group comparisons were conducted using the independent *t*-test for continuous data and the Chi-square test or Fisher’s exact test for categorical data. Within-group comparisons (preoperative, immediate postoperative, and 2-year postoperative) were conducted using the paired *t*-test or the Wilcoxon signed-rank test. Receiver operating characteristic (ROC) curve analysis was performed to determine the optimal cut-off values of C7SVA and PI–LL mismatch associated with L5–S1 disc degeneration. A *p*-value < 0.05 was considered statistically significant.

## 3. Results

### 3.1. Demographic Data

Of the 112 DSI patients included in the study, 64 (57.1%) had been treated with L5 laminar hook, while 48 (42.9%) were not. The average age in the two groups was 73.5 ± 7.19 and 72.86 ± 6.20 years, respectively, and most were women (Group I: 28/64 vs. Group II: 20/48). There was no significant difference between the body mass index (BMI) and bone mineral density (BMD) in either group (BMI: *p* = 0.114; BMD: *p* = 0.625, respectively). For both Groups I and II, the upper instrumented vertebra (UIV) was predominantly located at T11 (Group I: *n* = 28, 43.8% vs. Group II: *n* = 16, 33.3%) or T12 (Group I: *n* = 22, 34.4% vs. Group II: *n* = 24, 50%). The primary diagnoses leading to previous surgical planning were similar between the two groups: multi-level spinal stenosis (Group I: 50/64, 75% vs. Group II: 36/48, 75%), multiple-level fractures (Group I: 8/64, 12.5% vs. Group II: 6/48, 12.5%), iatrogenic flatback (Group I: 2/32, 6.3% vs. Group II: 4/48, 8.3%), and post-traumatic kyphosis (Group I: 2/64, 3.1% vs. Group II: 2/48, 4.2%); there was no significant difference between the two groups (*p* = 0.546) in this respect. Most of the patients had a PI > 45° (94/112, 84%); there was no significant difference between the two groups in this respect (*p* = 0.411) (Table 1).

### 3.2. Disc Status

**Figure 1:**
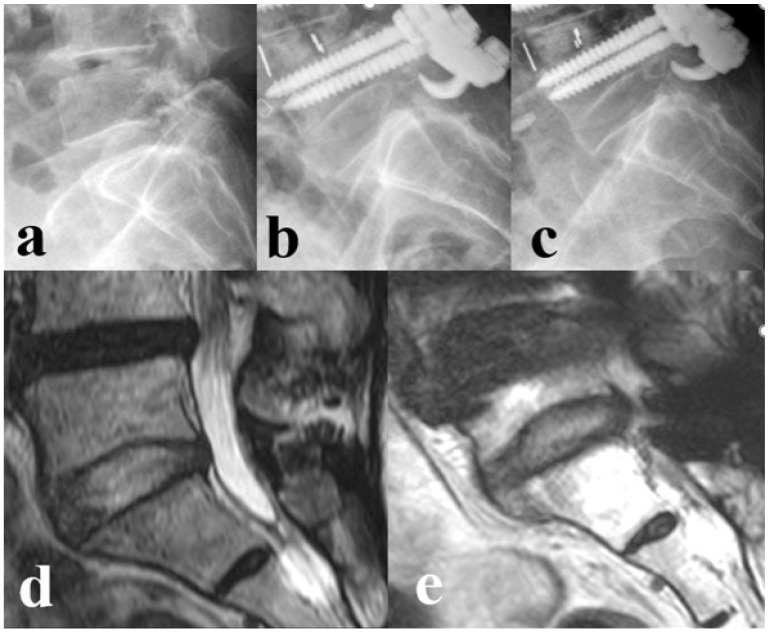
Standing lateral radiographs and MRI status in L5–S1 level of a 67-years old female. (**a**) Preoperative L5–S1 status. (**b**) Immediate postoperative L5–S1 status. (**c**) 2-year follow-up L5–S1 status, shows that there is little decrease in disc height compared to the immediate postoperative status. (**d**) Preoperative L5–S1 status, showing Pfirrmann grade 1. (**e**) 2-year follow-up L5–S1 status, showing Pfirrman grade 2.

Immediate postoperative status of the L5–S1 discs was considered healthy (Pfirrmann grade 1, 2, or 3) for both groups (100%). Two years post-surgery, a significantly higher number of patients in Group II had developed L5–S1 disc degeneration (*n* = 36/48, 75%) when compared to that in Group I (*n* = 14/64, 21.9%) (*p* < 0.001) (Figure 1, Table 2).

### 3.3. Sagittal Spinopelvic Parameters

**Figure 2:**
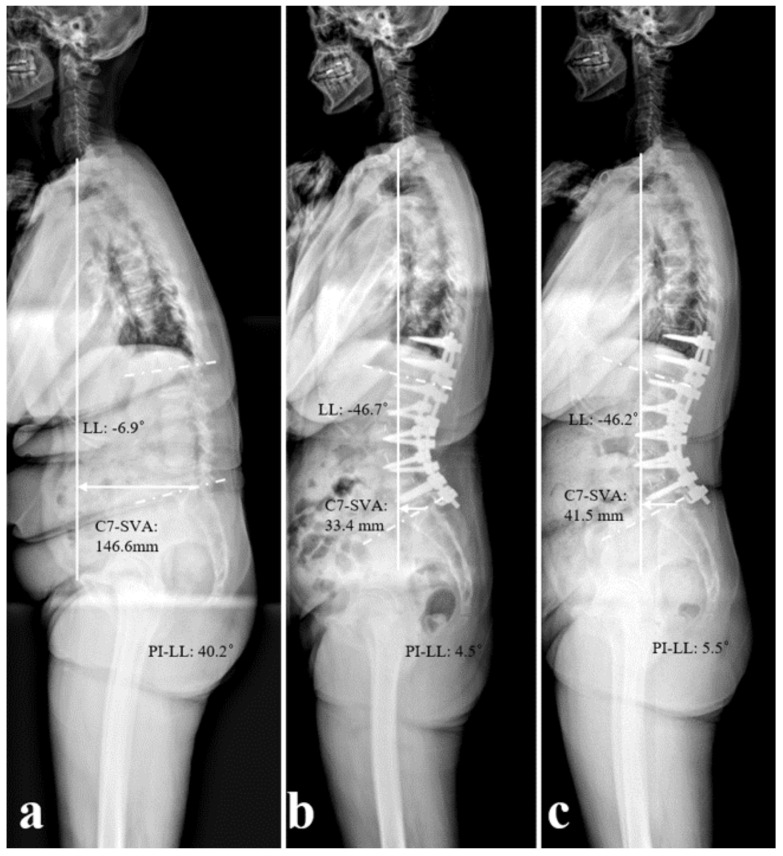
Standing lateral radiographs of a 63-year-old female patient who underwent long-level surgery from T11 to L5, with an L5 laminar hook. (**a**) Preoperative status, showing significant sagittal imbalance, with a C7-SVA of 146.6 mm, LL of −6.9°, and PI-LL of 40.2°. (**b**) Postoperative status, showing satisfactory correction; the C7-SVA was 33.4 mm, the LL was −46.7°, and the PI-LL was 4.5°. (**c**) Two years after surgery, the patient had maintained good sagittal alignment.

The patients in Group I had more severe preoperative sagittal imbalance than those in Group II (C7SVA: 174.6 ± 45.5 vs. 52.9 ± 65.1 mm, *p* = 0.011; LL: −11.6 ± 19.3 vs. −22.8 ± 23.6°, *p* = 0.029; PI-LL: 44.4 ± 17.5 vs. 29.1 ± 21.2°, *p* = 0.041). For both groups, radiographic parameters had improved significantly post-surgery, and there was no significant loss of correction when assessed at the 2-year follow-up. The degree of correction for C7SVA, LL, and PI-LL mismatch in Group I was significantly greater than that in Group II (C7SVA: −136.2 ± 68.6 vs. −29.3 ± 75.8 mm, *p* = 0.018; LL: −40.5 ± 15.4 vs. −25.4 ± 21.3°, *p* = 0.021; PI-LL: −40.5 ± 15.3 vs. −24.8 ± 21.4°, *p* = 0.034) (Figure 2, Table 3).

### 3.4. Subgroup Analysis

Subgroup analysis of the patients in Group I, based on the L5–S1 disc status after 2 years of follow-up, failed to identify significant differences for most demographic data. However, the patients in Group A (preserved disc, *n* = 50) had a significantly lower BMI than those in Group B (exacerbated disc, *n* = 14) (24.11 ± 3.52 vs. 27.94 ± 4.39; *p* = 0.038) (Table 4).

**Figure 3:**
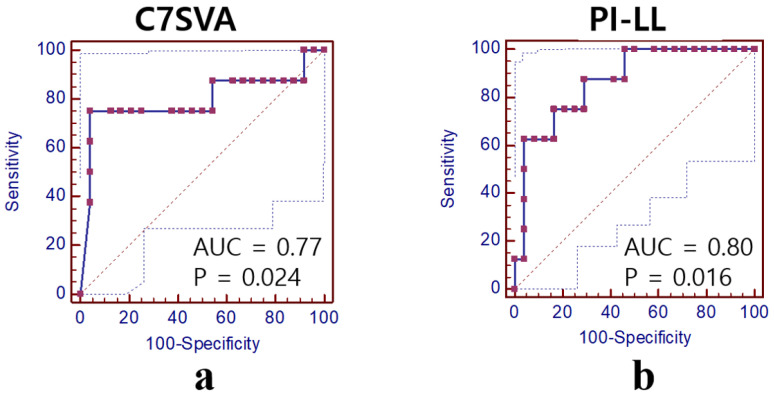
Receiver-operator characteristic curve (ROC) analyses for the prediction of preoperative C7SVA and PI-LL in Group I. (**a**) C7SVA: area under the curve (AUC) = 0.77, *p* = 0.024. (**b**) PI–LL: AUC = 0.80, *p* = 0.016.

Preoperatively, patients in Group B exhibited a more severe sagittal imbalance than those in Group A (C7SVA: 205.8 ± 109.4 vs. 158.9 ± 94.1 mm, *p* = 0.026; PI-LL: 51.9 ± 14.4 vs. 40.7 ± 18.1°, *p* = 0.039) (Table 5). ROC curve analysis demonstrated that both preoperative C7SVA and PI–LL mismatch were significant predictors of postoperative L5–S1 disc degeneration. The optimal cut-off value for C7SVA was 15.8 cm (AUC = 0.77, 95% CI = 0.35–0.97, *p* = 0.024), with 75.0% sensitivity and 95.8% specificity. For PI–LL mismatch, the optimal cut-off was 40.8° (AUC = 0.80, 95% CI = 0.47–0.99, *p* = 0.016), showing 90.5% sensitivity and 70.8% specificity. The positive predictive value (PPV) and negative predictive value (NPV) were approximately 97% and 79% for C7SVA, and 76% and 88% for PI–LL mismatch, respectively (Figure 3, Table 6).

### 3.5. Assessment of the Reliability of Radiographic Parameters and Disc Status Measurements Using ICC

The ICC for intra-rater reliability was good to excellent for preoperative, immediate postoperative, and 2 years post-surgery follow up radiographic parameter measurements (preoperative: 0.83 to 0.95; immediate postoperative: 0.86 to 0.95; 2 years post-surgery: 0.84 to 0.96, respectively). The ICC for inter-rater reliability was also good to excellent for preoperative, immediate postoperative, and 2 years post-surgery follow-up radiographic parameter measurements (preoperative: 0.81 to 0.95; immediate postoperative: 0.84 to 0.97; 2 years post-surgery: 0.86 to 0.94, respectively). And the ICC for intra-rater reliability was good to excellent for preoperative and 2 years post-surgery follow-up disc status measurements (preoperative: 0.87 to 0.97; 2 years post-surgery: 0.85 to 0.95, respectively).

## 4. Discussion

The laminar hook was first introduced as an adjunct to the pedicle screw and rod system [3,4,5] and is commonly used to treat thoracolumbar fractures, as it allows for short-segment pedicle fixation while preserving the spinal motion segments [10]. The application of a supra-laminar hook for the proximal segment of spinal fusion can protect the superior pedicle screws and reduce the risk of disease in the adjacent segment by gradually distributing stress between fused and unfused segments. This method can be used to prevent proximal junctional failure by allowing a ‘soft landing’ when used in the UIV. On the other hand, the role of a laminar hook in the distal segment is to protect the distal pedicle screws [10,11]. Sun et al. [12] reported that augmenting the pedicle screws with infra-laminar hook in the same vertebra at the distal construct improved the pullout strength of the screws by 21%. However, few studies have investigated the effects of laminar hooks in terms of correcting sagittal imbalance and preventing disc degeneration in patients with DSI. In this study, we aimed to determine the effect of the L5 laminar hook on sagittal spinopelvic parameters and L5–S1 disc degeneration when performing long-level fusion terminating at L5 in patients with DSI.

In the current study, the mean age of patients in both groups was over 70 years, with relatively healthy intervertebral discs at the L5–S1 level and without severe osteoporosis. And most patients were diagnosed with spinal stenosis accompanied by flatback deformity. Given that elderly patients generally exhibit lower levels of physical activity compared to younger individuals, we opted to use a laminar hook or end up at L5 to correct sagittal imbalance during preoperative planning, rather than expanding to S1 or pelvic. The fusion levels were comparable between the two groups, and as a result, the perioperative processes were largely similar. A previous study [13] analyzed the effects of posterior multi-segmented spinal hooks on sagittal correction in patients with idiopathic scoliosis; authors successfully preserved and restored normal LL by applying spinal hooks. Another study showed that the application of a hook could obtain more corrective angles because the laminar hook-rod interface in the distal segment serves as a cantilever beam to achieve better sagittal correction [11]. Based on previous studies, we applied the L5 laminar hook for surgical treatment of patients with more severe sagittal imbalance (a larger C7SVA, lower LL, and worse PI-LL mismatch). Postoperatively, the patients in both of our groups achieved significant sagittal balance correction, an outcome that was maintained over the 2 years follow-up period. In addition, we observed that the patients in Group I achieved a greater degree of correction; this was because these patients had more severe preoperative sagittal imbalance. Although we were unable to establish the superiority of the laminar hook for surgical correction, our results were consistent with those of previous studies showing that the L5 laminar hook effectively maintained corrected sagittal alignment in patients with severe sagittal imbalance.

Disc degeneration, retrolisthesis, or stenosis in adjacent segments are well-known risk factors for adjacent segmental degeneration (ASD) [14,15]. Cannizzaro Et Al. [7] reported the risk factor of ASD according to Pfirrmann grade in disc degeneration of adjacent segments, and based on this, we classified Pfirrmann grade 1, 2, and 3 as ‘healthy status’ and grade 4 and 5 as ‘degenerated status’ in this study. Previous studies [16,17] reported that the rates of post-surgical L5–S1 disc degeneration were approximately 58% and 69% following long-level surgery that stopped at L5. In the present study, 75% of patients in Group II experienced post-surgical L5–S1 disc degeneration (from ‘healthy status’ to degenerated status’) within two years of surgery, which was a higher proportion than that reported in previous studies. We believe that our worse results were due to the fact that the patients in our study were elderly (72.86 ± 6.20 years of age), had a high BMI (26.86 ± 4.67 kg/m^2^), and had a long fusion level; these are all known preoperative risk factors for disc degeneration [18]. Meanwhile, Group I patients who had an L5 laminar hook applied, 78% of patients did not develop L5–S1 disc degeneration 2 years after surgery. Previous studies demonstrated that the addition of laminar hooks in a pedicle screw-rod construct increased pullout resistance and contributed to load sharing or stress distribution between the instrumented segment and the adjacent level without affecting the segments with normal motion [10,11,19]. In addition, laminar hooks increase the bone–implant contact surface area, thus facilitating resistance against dorsally directed forces at the distal segment [19]. Collectively, our results also showed that the L5 laminar hook prevented subsequent L5–S1 disc degeneration when used for long-level fusion.

Through our analysis, we found that the L5 laminar hook could effectively prevent degeneration of the distal adjacent disc; however, exacerbation was detected in seven patients (21%). In subgroup analysis within Group I (in which a laminar hook was applied), the patients in Group B (exacerbated disc) had a significantly higher BMI than those in Group A (preserved disc). This suggests that the benefit of laminar hooks for the prevention of L5–S1 degeneration is limited to patients with higher BMI levels. Furthermore, our comparison of sagittal spinopelvic parameters revealed significantly greater C7SVA and worse PI-LL mismatch values for patients in Group B. ROC curve analysis indicated that the cutoff threshold values for C7SVA and PI-LL mismatch were 15.8 cm and 40.8°, respectively. These data imply that in patients with high BMI or severe sagittal imbalance (high C7SVA or PI-LL mismatch), fusion extension to the S1 or pelvis may be considered rather than application of the L5 laminar hook.

In the present study, we investigated the utilization of laminar hooks in the management of patients with DSI and evaluated the impact of this technique on sagittal balance correction and its potential ability to prevent disc degeneration in the distal segment. However, there were several limitations to our study that need to be considered. First, this study was a retrospective study conducted at a single institution with a relatively small sample size. Therefore, a large-scale randomized controlled trial is needed to prove the clinical indication of the L5 laminar hook, including the cutoff values of C7SVA and PI-LL mismatch suggested in this study. First, this study was a retrospective study conducted at a single institution with a relatively small sample size. Therefore, a large-scale randomized controlled trial is needed to prove the clinical indication of the L5 laminar hook, including the cutoff values of C7SVA and PI-LL mismatch suggested in this study. Second, there may be selection bias because we empirically applied laminar hooks to patients with severe DSI and included them in this study. Third, we were unable to acquire data relating to the complications of patients who had been treated with laminar hooks from the available medical records. Previous studies have reported the occurrence of laminar fractures and spinal stenosis, especially when two laminar hooks were applied; furthermore, the loss of integrity in the ligamentum flavum can lead to sagittal decompensation [12]. Third, we did not evaluate the coronal plane, because we excluded the patients diagnosed with degenerative lumbar scoliosis, which would change our preoperative planning. Furthermore, a previous meta-analysis study demonstrated that long fusion terminating at L5 or the sacrum was similar in scoliosis correction; moreover, similarities were observed in the complication rate, revision rate, and improvement in pain and disability in patients with adult spinal deformity [20]. Finally, patient-reported outcomes, such as the Oswestry disability index or visual analog scale, were not compared between the groups. A comparison of the health-related quality of life outcomes of patients with L5 laminar hook after surgery may provide a better understanding of the instrumental role. Despite these limitations, the strength of this study is that we analyzed the clinical outcome of using the L5 laminar hook when performing long-level instrumented and fusion surgery terminating at L5 in patients with adult spinal deformity. Additionally, this study clearly demonstrated the advantages of the L5 laminar hook by long-term follow-up of the fate of the L5–S1 disc level, thereby providing a good clinical analysis for many spine surgeons considering long-level fusion terminating at L5.

## 5. Conclusions

The use of an L5 laminar hook for the surgical correction of DSI can allow spinal surgeons to spare the sacropelvic region during long-level fusion. Furthermore, laminar hooks can provide an effective degree of correction in sagittal balance and may potentially prevent the progression of L5–S1 disc degeneration. Our data suggest that patients with a high BMI and severe sagittal imbalance may not benefit from L5 laminar hooks. The threshold for application of L5 laminar hooks was a preoperative C7SVA and PI-LL mismatch of <15.8 cm and 40.8°, respectively.

## Figures and Tables

**Table 1:** Demographic data of Group I and Group II.

	Group I (Hook+, *n* = 64)	Group II (Hook−, *n* = 48)	*p*-Value
Age at surgery (years)	73.5 ± 7.19	72.86 ± 6.20	0.128
Sex (Female/Male)	28:4	20:4	0.269
BMI (kg/m^2^)	25.05 ± 3.98	26.86 ± 4.67	0.114
BMD (T-score)	−1.24 ± 1.14	−1.03 ± 2.15	0.625
UIV level (*n*, %)			0.125
T9	2 (3.1%)	2 (4.2%)	
T10	12 (18.7%)	6 (12.5%)	
T11	28 (43.8%)	16 (33.3%)	
T12	22 (34.4%)	24 (50%)	
Preoperative diagnosis (*n*, %)			0.546
Multi-seg. Spinal stenosis with sagittal imbalance	50 (78.1%)	36 (75%)	
Multiple-level fractures	8 (12.5%)	6 (12.5%)	
Iatrogenic flatback	4 (6.3%)	4 (8.3%)	
Post-traumatic kyphosis	2 (3.1%)	2 (4.2%)	
Pelvic incidence (*n*, %)			0.411
≤45°	10 (15.6%)	8 (16.6%)	
>45° but ≤60°	30 (46.9%)	20 (41.7%)	
>60°	24 (37.5%)	20 (41.7%)	

Values are presented as mean ± standard deviation. BMI, body mass index; BMD, bone mineral density; UIV, upper instrumented vertebra; T, thoracic; Seg, segmental.

**Table 2:** Progressed degeneration of L5–S1 between Group I and Group II.

	Group I (Hook+, *n* = 64)	Group II (Hook−, *n* = 48)	*p*-Value
Degeneration Grade of L5–S1 (*n*, %)			
Preoperative			1.000
Pfirrmann grade 1, 2 or 3 (healthy)	64 (100%)	48 (100%)	
Pfirrmann grade 4 or 5 (degenerated)	0	0	
Follow-up at 2 years			<0.001 *
Pfirrmann grade 1, 2 or 3 (healthy)	50 (78.1%)	12 (25.0%)	
Pfirrmann grade 4 or 5 (degenerated)	14 (21.9%)	36 (75.0%)	

* A statistically significant difference between both groups (*p* < 0.05). Values are presented as mean ± standard deviation.

**Table 3:** Sagittal spinopelvic parameters of Group I and II.

Parameter		Group I (Hook+, *n* = 64)	Group II (Hook−, *n* = 48)	*p*-Value
C7 SVA (mm)	Preop.	174.6 ± 45.5	52.9 ± 65.1	0.011 *
	Immed. postop.	38.1 ± 45.3	23.8 ± 35.4	0.412
	2 years postop.	49.4 ± 33.8	54.5 ± 64.3	0.584
	Change	−136.2 ± 68.6	−29.3 ± 75.8	0.018 *
TK (°)	Preop.	13.8 ± 14.5	8.2 ± 18.5	0.333
	Immed. postop.	24.8 ± 11.7	23.3 ± 14.6	0.611
	2 years postop.	26.7 ± 10.8	27.9 ± 16.3	0.584
	Change	10.9 ± 9.5	15.1 ± 14.4	0.219
TLK (°)	Preop.	16.9 ± 16.2	12.5. ± 14.2	0.398
	Immed. postop.	2.6 ± 10.4	0.7 ± 5.7	0.454
	2 years postop.	7.4 ± 12.1	5.3 ± 6.6	0.433
	Change	−14.3 ± 14.9	−16.2 ± 15.5	0.414
LL (°)	Preop.	−11.6 ± 19.3	−22.8 ± 23.6	0.029 *
	Immed. postop.	−52.1 ± 10.3	−48.2 ± 17.7	0.299
	2 years postop.	−46.9 ± 11.2	−43.3 ± 20.9	0.322
	Change	−40.5 ± 15.4	−25.4 ± 21.3	0.021 *
PT (°)	Preop.	26.8 ± 8.6	24.4 ± 9.7	0.428
	Immed. postop.	18.6 ± 7.9	18.1 ± 9.9	0.644
	2 years postop.	22.6 ± 8.1	21.4 ± 9.2	0.521
	Change	−8.2 ± 7.8	−6.3 ± 4.8	0.447
SS (°)	Preop.	26.5 ± 10.2	29.1 ± 7.9	0.341
	Immed. postop.	38.2 ± 9.3	34.7 ± 7.6	0.331
	2 years postop.	34.1 ± 8.1	31.2 ± 7.2	0.421
	Change	7.6 ± 7.9	5.6 ± 5.7	0.457
PI-LL (°)	Preop.	44.4 ± 17.5	29.1 ± 21.2	0.041 *
	Immed. postop.	3.9 ± 9.3	4.3 ± 6.6	0.551
	2 years postop.	−10.51 ± 5.5	−15.42 ± 9.72	0.551
	Change	−40.5 ± 15.3	−24.8 ± 21.4	0.034 *

* A statistically significant difference between both groups (*p* < 0.05). Values are presented as mean ± standard deviation. SVA, sagittal vertical axis; TK, thoracic kyphosis; TLK, thoracolumbar kyphosis; PI, pelvic incidence; PT, pelvic tilt; LL, lumbar lordosis.

**Table 4:** Demographic data of Group A and B in Group I.

	Group A (Preserved, *n* = 50)	Group B (Exacerbated, *n* = 14)	*p*-Value
Age at surgery (years)	72.86 ± 6.43	74.13 ± 8.28	0.187
Gender (Female/Male)	22:3	6:1	0.245
BMI (kg/m^2^)	24.11 ± 3.52	27.94 ± 4.39	0.038 *
BMD (T-score)	−1.25 ± 3.52	−1.26 ± 1.20	0.523
Preoperative diagnosis (*n*, %)			0.312
Multi-seg. Spinal stenosis with sagittal imbalance	40 (80.0%)	12 (85.7%)	
Multiple-level fractures	6 (12.0%)	2 (14.3%)	
Iatrogenic flatback	2 (4.0%)	0	
Post-traumatic kyphosis	2 (4.0%)	0	
Preop. L5–S1 Deg. grade (*n*, %)			0.891
Pfirrmann grade 1, 2, or 3	50 (100%)	14 (100%)	
Pfirrmann grade 4 or 5	0	0	

* A statistically significant difference between both groups (*p* < 0.05). Values are presented as mean ± standard deviation. BMI, body mass index; BMD, bone mineral density; T, thoracic; Deg, degenerative.

**Table 5:** Sagittal spinopelvic parameters of Group A and B.

Parameter		Group A (Preserved Disc)	Group B (Exacerbated Disc)	*p*-Value
C7 SVA (mm)	Preop.	158.9 ± 94.1	205.8 ± 109.4	0.026 *
	Immed. postop.	34.7 ± 36.1	45.8 ± 60.1	0.312
	Change	−124.2 ± 85.7	−160.0 ± 77.8	0.037 *
TK (°)	Preop.	12.1 ± 9.9	14.7 ± 16.5	0.511
	Immed. postop.	19.6 ± 8.2	27.4 ± 12.5	0.227
	Change	7.5 ± 8.1	12.7 ± 10.8	0.331
TLK (°)	Preop.	14.5 ± 22.4	18.7 ± 15.3	0.317
	Immed. postop.	1.4 ± 10.3	4.1 ± 10.9	0.385
	Change	−13.1 ± 10.8	−14.6 ± 9.8	0.554
LL (°)	Preop.	−15.1 ± 16.9	−8.8 ± 23.1	0.167
	Immed. postop.	−53.9 ± 10.8	−48.5 ± 8.8	0.221
	Change	−38.8 ± 22.7	−39.7 ± 22.7	0.548
PT (°)	Preop.	28.1 ± 8.5	26.9 ± 9.1	0.531
	Immed. postop.	18.3 ± 8.4	19.3 ± 7.5	0.544
	Change	−10.8 ± 11.4	−7.6 ± 7.4	0.362
SS (°)	Preop.	25.4 ± 10.9	27.4 ± 9.1	0.487
	Immed. postop.	37.6 ± 8.1	36.4 ± 11.9	0.514
	Change	12.2 ± 7.9	9.1 ± 10.8	0.365
PI-LL (°)	Preop.	40.7 ± 18.1	51.9 ± 14.4	0.039 *
	Immed. postop.	2.2 ± 10.5	7.5 ± 8.1	0.287
	Change	−38.5 ± 12.8	−44.4 ± 10.8	0.044 *

* A statistically significant difference between both groups (*p* < 0.05). Values are presented as mean ± standard deviation. SVA, sagittal vertical axis; TK, thoracic kyphosis; TLK, thoracolumbar kyphosis; PI, pelvic incidence; PT, pelvic tilt; LL, lumbar lordosis.

**Table 6:** Receiver Operating Characteristic Analysis of C7SVA and PI-LL mismatch.

Parameter	Cut-Off	AUC (95% CI)	Sensitivity (%)	Specificity (%)	PPV (%)	NPV (%)
C7SVA (cm)	15.8	0.77 (0.35–0.97)	75.0	95.8	97.0	79.0
PI-LL (°)	40.8	0.80 (0.47–0.99)	90.5	70.8	76.0	88.0

C7SVA = C7 sagittal vertical axis; PI–LL = pelvic incidence–lumbar lordosis mismatch; CI = confidence interval; PPV = positive predictive value; NPV = negative predictive value.

## Data Availability

The datasets used and analyzed during the current study are available from the corresponding author on reasonable request.

## Abbreviations

The following abbreviations are used in this manuscript:

DSI	Degenerative sagittal imbalance
C7SVA	C7 sagittal vertical axis
LL	Lumbar lordosis
PI	Pelvic incidence
BMI	Body mass index
TK	Thoracic kyphosis
TLK	Thoracolumbar kyphosis
PT	Pelvic tilt
SS	Sacral slope
ROC	Receiver operating characteristic
